# Uptake and reaction to roundup ultra 360 SL in soybean seedlings

**DOI:** 10.2478/s11756-018-0092-8

**Published:** 2018-08-06

**Authors:** Agnieszka I. Piotrowicz-Cieślak, Łukasz Sikorski, Bożena Łozowicka, Piotr Kaczyński, Dariusz J. Michalczyk, Agnieszka Bęś, Barbara Adomas

**Affiliations:** 1 Department of Plant Physiology, Genetics and Biotechnology, Faculty of Biology and Biotechnology, University of Warmia and Mazury in Olsztyn, Oczapowskiego 1A, 10-718 Olsztyn, Poland; 2 Department of Environmental Toxicology, Faculty of Environmental Management and Agriculture, University of Warmia and Mazury in Olsztyn, Prawocheńskiego 17, 10-720 Olsztyn, Poland; 3 Plant Protection Institute – National Research Institute, Laboratory of Pesticide Residues, Chełmońskiego 22, 15-195 Białystok, Poland

**Keywords:** Biogenic amines, Free radicals, Enzyme activity, Glyphosate content

## Abstract

Due to the widespread and frequent use of Roundup Ultra 360 SL in crops production, the active substance glyphosate is often present (in the soil or in post-harvest remnants) and may be toxic to plants, including the non-target species. The aim of the current study was to determine the sensitivity of young soybean seedlings to glyphosate in concentrations ranging from 0 to 10 μM. It was demonstrated that the seedlings take small quantities of soil glyphosate up. More of the active substance was found in the shoots than in the roots. From the doses applied, the plant absorbs up to 4% of soil glyphosate, while over 96% remains in the soil. This suggests that only 4% of glyphosate taken up from the soil affects plant seedling development and water management. It modifies the contents of the biogenic amines cadaverine and putrescine as well as the activity of enzymes involved in their biosynthesis, i.e. ornithine decarboxylase and lysine decarboxylase. The free radical content of the roots increased with increasing herbicide doses and time of exposure. The main enzyme involved in the rapid removal of free radicals was superoxide peroxidase, activated by the herbicide treatment, while catalase was not significantly stimulated.

## Introduction

Highly toxic substances are used in plant protection and deliberately introduced into the agrocenoses. The presence of these undesirable compounds in food of plant origin is a crucial factor limiting its quality and safety. Particular attention is paid to chemical pollutants which include pesticides (Łozowicka et al. 2016). In modern agriculture, chemicals are routinely used for the protection of crops against pests, pathogens and weeds. Therefore, the plant protection products, apart from their beneficial effect on plant production, may have adverse effects on plants and the environment (Aktar et al. 2009). Glyphosate [N-(phosphonomethyl)glycine; GlyPh] is the active substance of a number of herbicides used to control mono- and dicotyledonous weeds in agriculture, vegetable and fruit farming, as well as superfluous vegetation in water bodies. Even though it has been used for almost 40 years, its heyday only began with the advance of genetic engineering. The cultivation of the so called Roundup-ready varieties has resulted in establishing Roundup as the most widely used herbicide in the world. In recent years its use has been on the increase, even in those countries that regulate GM plants very sternly. In Germany for example from 1999 to 2010 it increased by 100%. The mechanism of GlyPh toxic action involves the inhibition of the shikimate pathway, i.e. one of the main metabolic pathways in plants (Martini et al. 2012). GlyPh destroys troublesome weeds; it does not, however, inhibit the growth of the Roundup-ready crops which allows it to be used as a perfectly selective herbicide on a large scale. Due to the rapid and effective action of GlyPh, as well as the absence of a shikimate pathway in humans and the resulting high selectivity of this pesticide, it is favoured by farmers and considered relatively safe for humans. On the other hand, there are some reports suggesting that GlyPh in addition to inhibiting the key enzyme of the shikimate pathway (5-enolpyruvylshikimate 3-phosphate synthase), may also affect other metabolic processes in plants (Naka et al. 2010; Yang et al. 2010), in particular those related to stress responses. Biogenic amines are molecules known to participate in plant stress responses, although their role has not been studied very extensively (Baciak et al. 2017).

Examples of genetically modified plants which exhibit high resistance to GlyPh include cotton, corn and soybean (Steinmann et al. 2012). GlyPh has an affinity for water molecules, hence its high water solubility (from 10 to 15.7 g × L^−1^at a temperature of 25 °C) (Modesto and Martinez 2010). On the other hand, since it is practically insoluble in organic solvents, Pieniążek et al. (2004) claim that GlyPh should not be dissolved in fats and that, consequently, it should not be accumulated in animal tissues. Sandrini et al. (2013) have a different view on this topic and claim that certain organisms may accumulate N-(phosphonomethyl)glycine. Watts (2012), however, demonstrates that GlyPh and its metabolite aminomethylphosphonic acid exhibit medium persistence in the environment and that they are even detected in rainwater. What is more, those substances are able to accumulate in surface waters and in soil, which results from GlyPh affinity to the mineral components of soil. A study carried out in the Buenos Aires area found that GlyPh was present in significant quantities in soil (from 0.5 to 5 mg × kg^−1^) and in water (from 0.1 to 0.7 mg × L^−1^) (Peruzzo et al. 2008).

Discussion about GlyPh intensified in recent years when two completely different opinions on its harmful effects were expressed. The International Agency for Research on Cancer, which is a part of the World Health Organization (IARC 2015), demonstrated that GlyPh may contribute to cancer incidence, while according to the European Food Safety Authority, an advisory body to the European Commission, GlyPh has no adverse effects on human health (Glyphosate 2015). The EU’s authorisation procedure for pesticides, however is one of the strictest in the world (FAQs 2016). The licence for GlyPh use expired on 1 July 2016 but was effectively extended till 31 December 2017 (Commission Implementing Regulation 2016).

Due to the widespread and frequent use of GlyPh for the production of a very wide range of crops this substance (either as soil contaminant or present in post-harvest remnants) may be toxic not only to the target weeds but also to non-target species. It was also demonstrated that P fertilisation applied 10–35 days prior to sowing considerably induces the action of GlyPh, manifested by damage to soybean plants (Bott et al. 2011). GlyPh is taken up by the roots of target plants (weeds) but it can also easily penetrate the roots of crops causing their dieback (Coupland and Casely 1979; Kremer and Means 2009; Laitinen and Rämö 2007; Tesfamariam et al. 2009; Boutin et al. 2014).

It was demonstrated that the sensitivity of plants to GlyPh largely depends on the species, variety and the phase of plant development. Even plants within one species differ in sensitivity to GlyPh (Piotrowicz-Cieślak et al. 2010). Therefore, it is important to understand the physiological consequences of GlyPh action at various stages of growth and development, particularly in very young plants. Despite the considerable number of studies concerning GlyPh, the effects of this substance on the biogenic amine biosynthesis in soybean seedlings are still unknown. The aim of this study was, therefore, to analyze the metabolic responses of soybean seedlings to GlyPh at the early stages of growth, i.e. till development of the first true leaf.

## Material and methods

### Phytotoxkit test

Soybean seeds (*Glycine max* (L.) Merr) cv. Mazowia were germinated for 9 days under controlled lighting and temperature conditions (8 h (16 °C – night) and 16 h (20 °C, 140 μmol photons m^−2^ × s^−1^ PAR – day) in Phytotoxkit plates (MicroBio Test, Inc., Belgium) filled with a natural quartz sand with the grain size of 0.8–1.2 mm). Each culture was watered with 27 ml of distilled water (control) or 27 ml of Roundup Ultra 360 SL aqueous solutions (Monsanto, Creve Coeur, Greater St. Louis, Missouri Poland), so as to obtain the following final concentrations of GlyPh: 3 μM, 7 μM, 10 μM. On the 9th day, percent seed germination, the length of roots and shoots and the fresh and dry mass content were measured according to ISTA (1999). The osmotic potential of the shoots was measured using the Plant Water Status Console Model 3000 (Soil Moisture Equipment Corp, Santa Barbara, CA, USA). Each test was carried out in fifteen replications.

Chemicals: Glyphosate, CAS Number: 1071–83-6 was used as isopropylamine salt of glyphosate, Roundup, 360 g × L^−1^ (Monsanto Poland).

### Biogenic amines assay

Biogenic amines (BA) content was determined in 9-days old soybean seedlings (roots and shoots). The plant material was homogenised with cold 5% hydrochloric acid (Bouchereau et al. 2000), next was shaken for 1 h and then centrifuged at 16000 g for 30 min at 4 °C. The supernatants were filtered through a nylon membrane-based (pore size 0.22 μm) syringe filter (Filter-Bio, Nantong City, China). The filtrate was analyzed by ion-exchange chromatography using amino acids analyzer AAA400 (Ingos, Prague, Czech Republic). BAs were separated at 76 °C on a 70 × 3.7 mm column filed with Ostion Lg ANB (Ingos, Prague, Czech Republic) and then eluted from the ion-exchange column with two pH 5.65 sodium citrate buffers with the addition of 1.0 and 2.6 M sodium chloride. The quality and quantity of the BAs were analyzed by post-column ninhydrin derivatization and photometry (λ = 570 nm). The BAs standards of Sigma Aldrich (St. Louis, Mo, USA) were used. Quantities of BAs were expressed as mean ± SD for 3–5 replications of each treatment.

### Enzyme activity of decarboxylases

The activity of the following BA biosynthesis enzymes was determined in roots and shoots of 9-days old soybean seedlings: S-adenosylmethionine decarboxylase (SAMDC, EC 4.1.1.50), arginine decarboxylase (ADC, EC 4.1.1.19), ornithine decarboxylase (ODC, EC 4.1.1.17), lysine decarboxylase (LDC, EC 4.1.1.18) and tyrosine decarboxylase (TDC, EC 4.1.1.25). The plant material (300 mg) was frozen in liquid nitrogen and homogenised at 4 °C in 1 ml extraction buffer (pH 8,0) containing 50 mM phosphate, 1 mM 2-mercaptoethanol, 50 μM pyridoxal phosphate (Chattopadhyay et al. 1997). The extracts were centrifuged with RCF 12000 g at 4 °C for 15 min. The supernatants were next transferred to the chromatography vials (1.5 ml) and the samples were shaken and incubated at 37 °C for 2 h. S-adenozylomethionine (15 mM, S-(5′-adenosyl)-L-methionine chloride dihydrochloride, **Merck**), l-arginine (40 mM, **(S)-2-amino-5-guanidinopentanoic acid, Merck**), l-ornithine ((S)-2,5-diaminopentanoic acid monohydrochloride, Merck), l-lysine (40 mM, **(*S*)-2,6-diaminocaproic acid, Merck**) and l-tyrosine (40 mM, **(S)-2-amino-3-(4-hydroxyphenyl)propionic acid, Merck**) were used as substrates for ODC, ADC, SAMDC, LDC and TDC, respectively. The reactions were stopped by cooling down the samples to 0 °C. The amounts of released CO_2_ (ppm) were measured with a multi-gas analyzer equipped with an infrared detector (MultiRAE IR One-to-Five Gas Monitor) using a non-dispersive infrared (NDIR) sensor). Decarboxylase activity was expressed in μmol CO_2_ released per gram protein during 1 min. The protein content was determined according to Lowry et al. (1951).

### LC-MS/MS analysis of GlyPh content/residues

Sample treatment was based on modified QuPPe protocols (Anastassiades et al. 2015). The roots and shoots (10 g) were homogenized with 10 mL of 0.5% formic acid in methanol-water mixture (1:1, *v/v*). The mixture was immediately shaken for 5 min, and centrifuged for 5 min at 5000 g. Finally, the solution was filtered through a PTFE filter (0.2 μm porosity), transferred into a glass vial and analysed by LC-MS/MS. Chromatographic analyses were performed using an Eksigent Ultra LC-100 (Eksigent Technologies, Dublin, CA, USA) equipped with an autosampler and a thermostatted column oven. The analytes were separated on the Obelisc N column (2.1 mm ×100 mm, 5 μm particle size) (Selic, Wheeling, USA).

The parameters for GlyPh, including declustering potential (DP), collision energy (CE), collision cell exit potential (CXP), retention time, and fragments of target compound are summarized in Table 1. Syshoot control, data acquisition, and data analysis were all carried out using Analyst software version 1.6.2 (AB Sciex Instruments, Foster City, CA).

**Table 1:** LC-MS/MS parameters

	Retention time (min)	MRM transition m/z	CE (V)	DP (V)	EP (V)	CXP (V)
Glyphosate – quantification	4.62	168 > 63	−32	−110	−5	−10
Glyphosate – confirmation 1		168 > 124	−54	−90	−5	−10
Glyphosate – confirmation 2		168 > 150	−44	−65	−5	−10

### Peroxidase activity

Extracts used to determine peroxidase (POD) activity were prepared on ice. The roots and shoots (100 mg) were homogenised in 2 ml extraction buffer (0.1 M Tris/HCl; pH 7, 8.75% polyvinylpyrrolidone, 0.1 M KCl, 0.28% Triton X-100). Samples were centrifuged for 30 min at 1300 g at 4 °C. The supernatant was passed through membrane filters with 0.45 μm pore diameter. The protein content in samples was determined with the Lowry et al. (1951) protein assay. POD activity was determined based on the spectrophotometric detection in mixture containing 100 μl 1% guaiacol, 1 ml 0.1 M phosphate buffer (pH 7.2), 20 μl supernatant and 50 μl 0.18% H_2_O_2_. The absorption rate increase was measured at room temperature at the wavelength λ = 470 nm. One Unit of activity corresponds to the oxidation of 1 μmole H_2_O_2_ by 1 mg protein during 1 min. Each POD activity analysis was carried out in five replications. The protein content was determined according to Lowry et al. (1951).

### Catalase activity

The roots and shoots (200 mg) were homogenized in 1 mL of 50 mM phosphate buffer (pH 7.0), containing 1% polyvinylpyrrolidone, 0.2 mM EDTA and 1% Triton X-100. Samples were centrifuged for 20 min at 12000 g at 4 °C. The reaction mixture contained 15 mM H_2_O_2_ in 50 mM phosphate buffer (pH 7.0) and 50 μL extract. Decrease of absorbance was measured at room temperature at the wavelength λ = 240 nm. One Unit decomposes 1 μmol H_2_O_2_ during 1 min at pH 7.0 at room temperature for 1 mg of protein. Each catalase activity analysis was carried out in five replications. The protein content was determined according to Lowry et al. (1951).

### Reactive oxygen species

Soybean seeds were germinated on soil with an addition of deionised water. After 9 days, the seedlings were transferred to new, sterile Phytotoxkit test plates filled with quartz sand supplemented with a 0, 3, 7 and 10 μM solution of GlyPh. After 0, 1 and 3 h, roots of the seedlings were incubated in the dark for 30 min in 0.1 M phosphate-buffered saline (PBS, pH 7.4), with 10 μM 2′,7′- dichlorodihydrofluorescein diacetate (H_2_DCF-DA). Subsequently, they were washed several times with a 0.1 M PBS solution at pH 7.4. H_2_DCF-DA is degraded in live cells by intracellular esterases to lipophobic dichlorofluorescein (DCF), which emits a fluorescent signal. DCF reacts with reactive oxygen species (ROS), as well as with reactive nitrogen species and with lipid peroxides. In the experiment, the following excitation and emission wavelengths were used: for H_2_DCF-DA – 488 nm and 515–565 nm, DCF esterase reaction product – 405 nm and 480–550 nm, respectively. Dual staining images were collected during sequential scanning of each fluorochrome. Images show an overlay projections (snapshots collected from Z-series images) produced from sequential scanning and LAS software (Leica Application Suite 2.0.2 build 2038).

### Statistical analysis

The complex standard uncertainties of $f(t)=\frac{1}{\mathrm{C}}$ were calculated as

$$ u(f)=\sqrt{{\left(\frac{\partial f}{\partial C}\right)}^2{u}^2(C)} $$

where *u*(*C*) denotes standard uncertainty of concentration and *u*(*C*) = *SD*. Standard deviation (SD) was determined for all measurements.

One-way analysis of variance (ANOVA) followed by Tukey’a comparison post-hoc test (*p* ≤ 0.01) was applied to evaluate differences between controls and treatments.

## Results

**Fig. 1:**
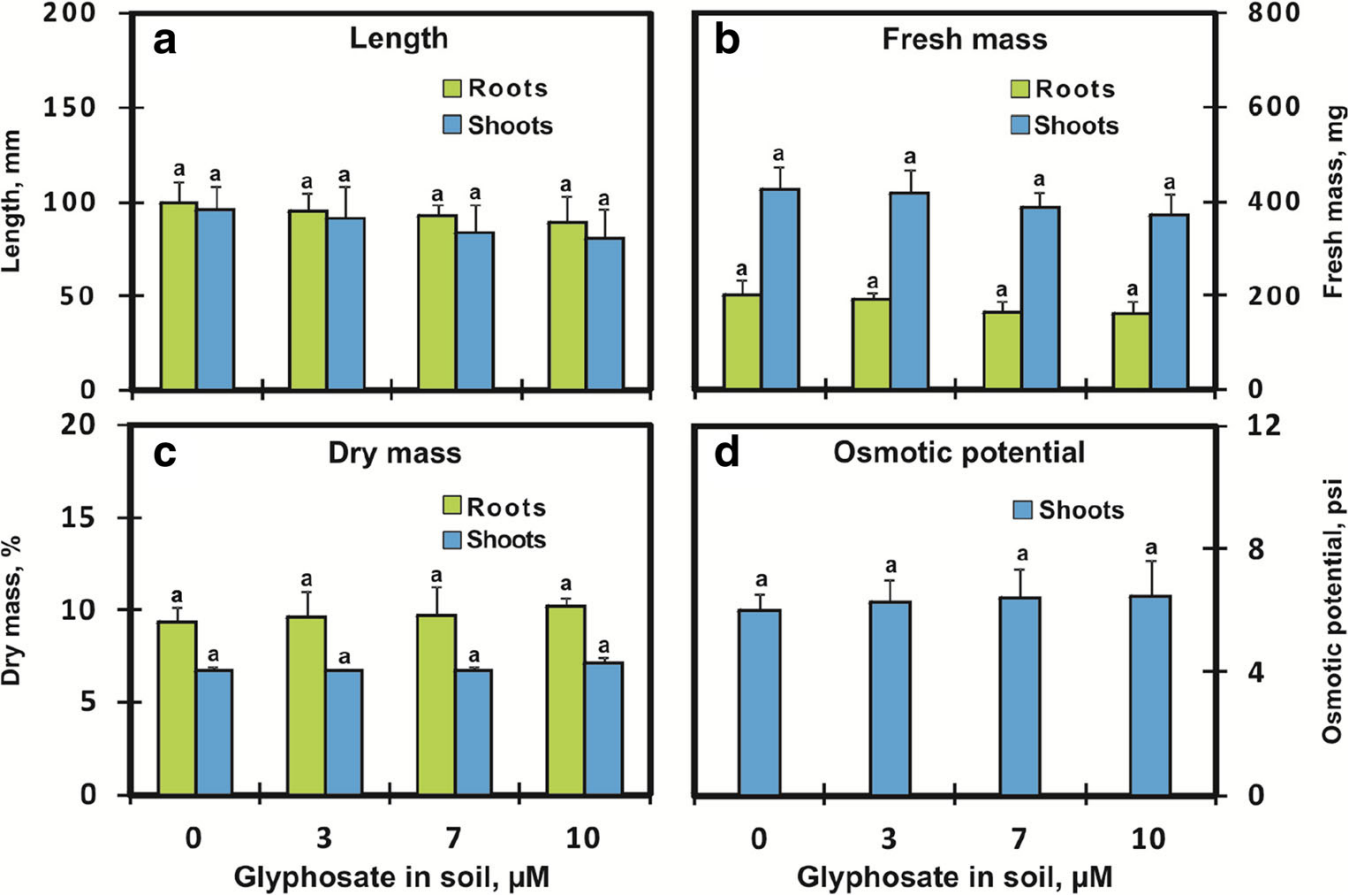
Length (**a**), fresh mass (**b**), dry mass (**c**), and osmotic potential (D) in roots () and shoots () of soybean seedlings growing for 9 days in soil contaminated with different GlyPh concentrations (0, 3, 7, 10 μM). Means with the same letter are not significantly different from each other (Tukey test, *p* ≤ 0.01)

After 9 days of exposure of soybean seeds to GlyPh (at concentrations 3 to 10 μM), neither inhibition of germination nor limiting of seedling growth (root and shoot length, fresh mass, dry mass), nor changes in osmotic potential were observed (Fig. 1).

**Fig. 2:**
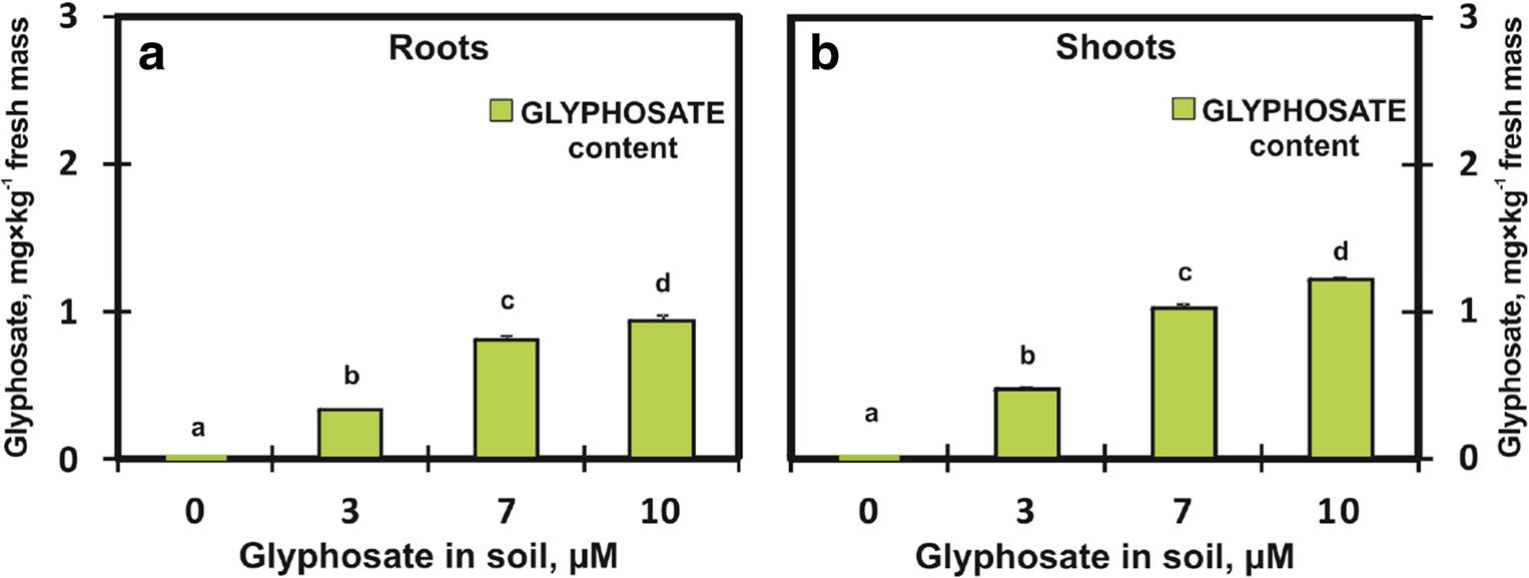
Glyphosate [mg×kg^−1^ fresh mass] content in roots (**a**) and shoots (**b**) of soybean seedlings growing for 9 days in soil contaminated with different GlyPh concentrations (0, 3, 7, 10 μM). Means with the same letter are not significantly different from each other (Tukey test, *p* ≤ 0.01)

GlyPh was taken up from soil by the roots and transported to the shoots as well. The roots of plants growing in soil contaminated with 3, 7, and 10 μM of the active substance (i.e. 3.84, 8.94, 12.78 mg × kg^−1^ of soil) contained 0.34, 0.82, and 0.94 mg GlyPh×(kg fresh mass)^−1^, while the shoots contained 0.48, 1.03, and 1.22 mg × kg^−1^ of the active substance concerned, respectively (Fig. 2). The total amounts of GlyPh taken up by an average root were 0.07, 0.13, 0.15 μg measured in seedlings subjected to the soil GlyPh at concentrations of 3, 7, and 10 μM, respectively. The shoots absorbed much higher amounts of GlyPh, i.e. 0.20, 0.40, and 0.45 μg at the soil GlyPh concentrations of 3, 7, and 10 μM, respectively.

**Fig. 3:**
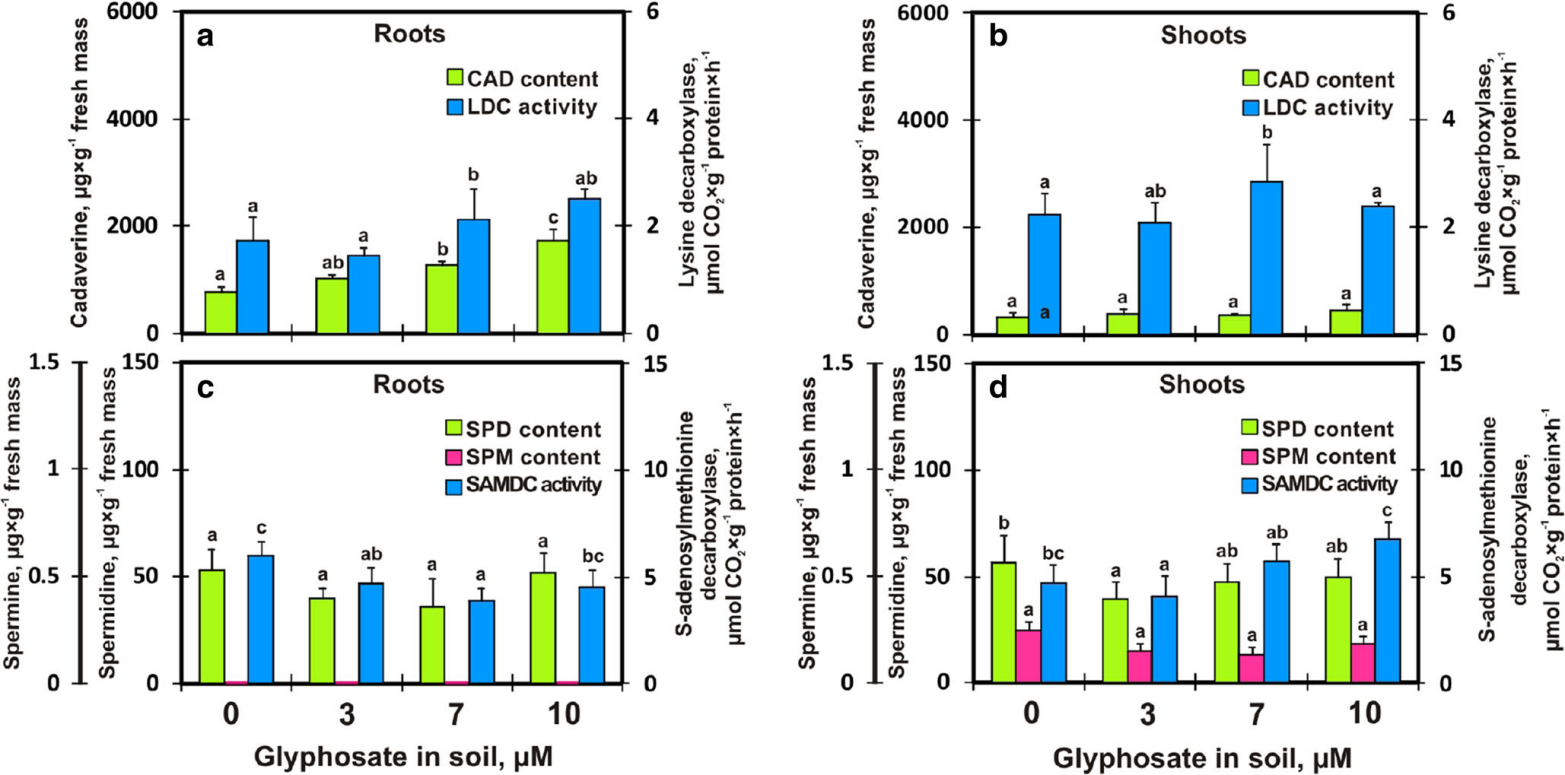
Biogenic amines contents and decarboxylase activity in roots (**a**, **c**) and shoots (**b**, **d**) of soybean seedlings growing for 9 days in soil contaminated with different GlyPh concentration (0, 3, 7, 10 μM Roundup Ultra 360 SL). Panel A and B: CAD – cadaverine content (), LDC – activity of lysine decarboxylase (); Panel C and D: SPD – content (), SPM – content (), SAMDC – S-adenosylmethionine decarboxylase activity (). Means with the same letter are not significantly different from each other (Tukey test, *p* ≤ 0.01)

The following BAs were detected in soybean seedlings treated with GlyPh: diamines, triamines and tetraamines. The diamines included putrescine (PUT) and cadaverine (CAD); triamines included spermidine (SPD); and tetraamines – spermine (SPM) and agmatine (AGM). In the roots, PUT, CAD and SPD were present, while in the shoots, in addition to the above BAs, SPM and agmatine occurred as well. Among all the BAs, CAD was by far the most abundant in both roots and shoots. In control roots the content of SPD was very low but it was considerable in shoots (56.81 μg × g^−1^ of fresh mass) (Fig. 3). In the roots of plants growing in soil contaminated with GlyPh at concentrations of 3, 7, and 10 μM, CAD content increased by 136%, 167 and 226%, respectively, while in the shoots it increased up to a maximum of 136% (Fig. 3). Unlike the increasing CAD content, SPD content in the roots decreased with an increase in soil GlyPh. As a result of soil application of GlyPh (7 μM) the SPD level in roots decreased from 52.99 μg × g^−1^ of fresh mass (in control plants) down to 35.82 μg × g^−1^ in GlyPh treatment (Fig. 3). LDC activity in the roots increased slightly and was highest at the highest GlyPh concentration applied. On the other hand, LDC activity in the shoots was at a similar level (Fig. 3). SAMDC activity slightly decreased in the roots, which resulted in an absence of SPM and in a slight increase in SPD content. On the other hand, in the shoots, SAMDC activity increased and SPM was present, while SPD content (similar to that in the roots) also increased (Fig. 3).

**Fig. 4:**
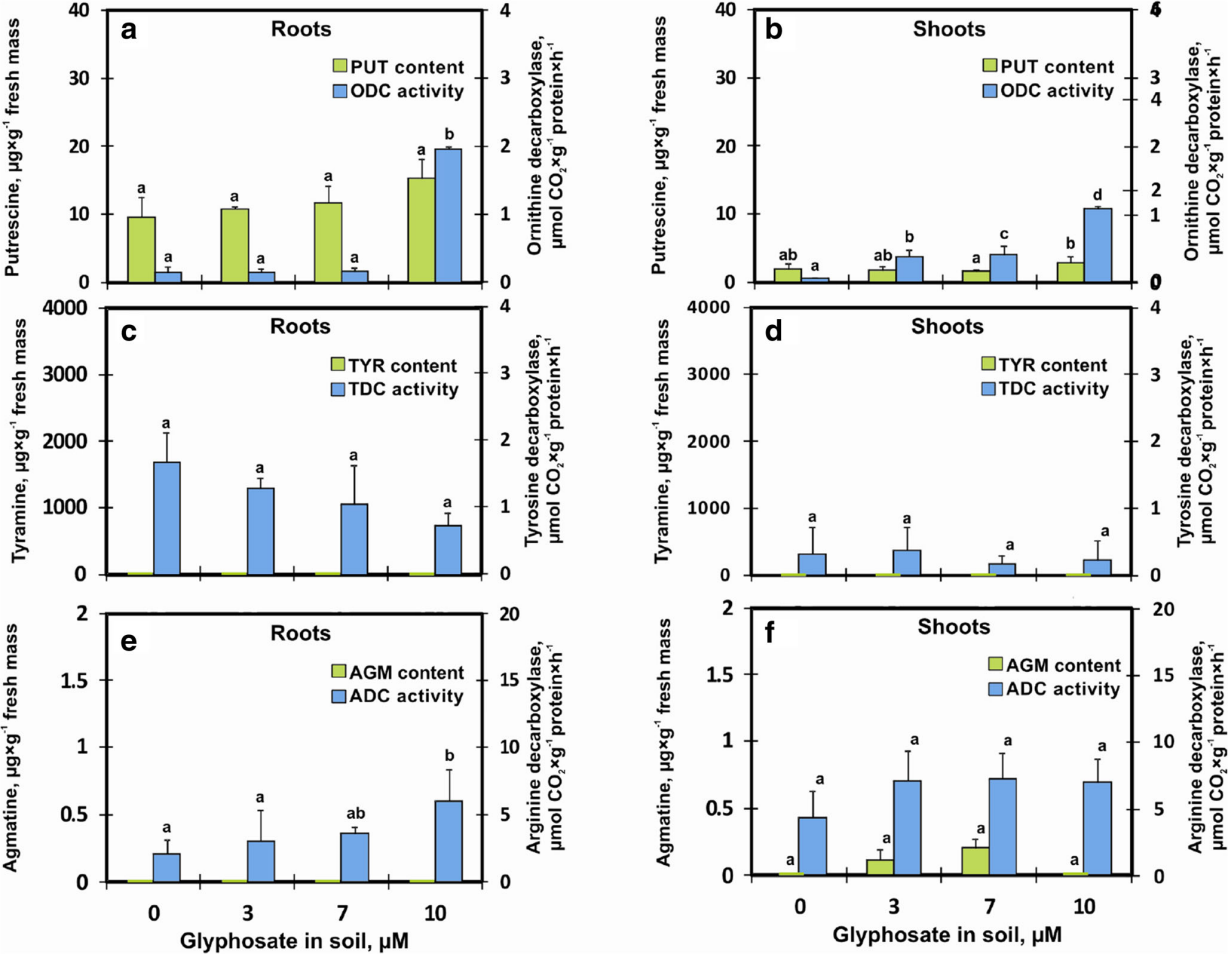
Biogenic amines contents and decarboxylase activity in roots (**a**, **c**, **e**) and in shoots (**b**, **d**, **f**) soybean seedlings growing for 9 days on soil contaminated with different GlyPh concentration (0, 3, 7, 10 ìM Roundup Ultra 360 SL). Panel A and B: PUT – putrescine content (), ODC – activity of ornithine decarboxylase (); Panel C and D: TYR – tyrosine content (), TDC – tyrosine decarboxylase activity (); Panel E and F: AGM – agmatine content (), ADC – arginine decarboxylase activity (). Means with the same letter are not significantly different from each other (Tukey test, *p* ≤ 0.01)

In the control roots and shoots, PUT was present at low concentrations; however, with an increase in GlyPh content, ODC activity increased, which resulted in an increase in PUT content (Fig. 4). Although AGM in the shoots was present in trace amounts, an increase in ADC activity in GlyPh treated plants was demonstrated. Similarly, TDC activity was found, although tyramine was not identified in 9-day-old seedlings (Fig. 4).

**Fig. 5:**
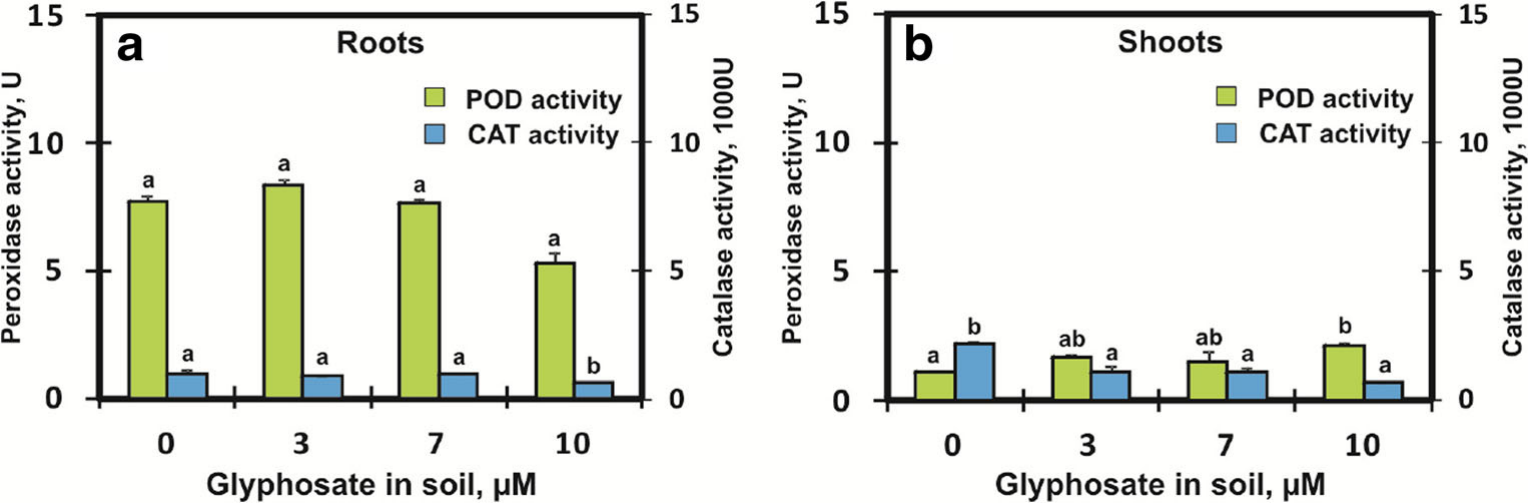
Peroxidase POD () and catalase CAT () activity in roots (**a**) and in shoots (**b**) of soybean seedlings growing for 9 days in soil contaminated with different GlyPh concentrations (0, 3, 7, 10 μM Roundup Ultra 360 SL). Means with the same letter are not significantly different from each other (Tukey test, *p* ≤ 0.01)

The activity of antioxidative enzymes, i.e. catalase (CAT) and peroxidase (POD) is presented in Fig. 5. POD activity in the control roots was seven times higher than in the shoots, however this difference was decreased at the highest GlyPh concentrations. An increase in the herbicide concentration from 7 to 10 μM decreased POD activity in the roots by 2.4 U compared to the control roots (7.72 U). In the shoots, an increase in POD activity from 1.08 U (control) to 2.14 U (10 μM) was observed (Fig. 5). CAT activity (in contrast to POD) decreased in both the roots and in the shoots. CAT activity was two times higher in control shoots (2.2 U) compared to control roots (0.97 U). CAT activities in roots and shoots at the highest GlyPh concentration were similar (0.62 U and 0.69 U, respectively) (Fig. 5).

**Fig. 6:**
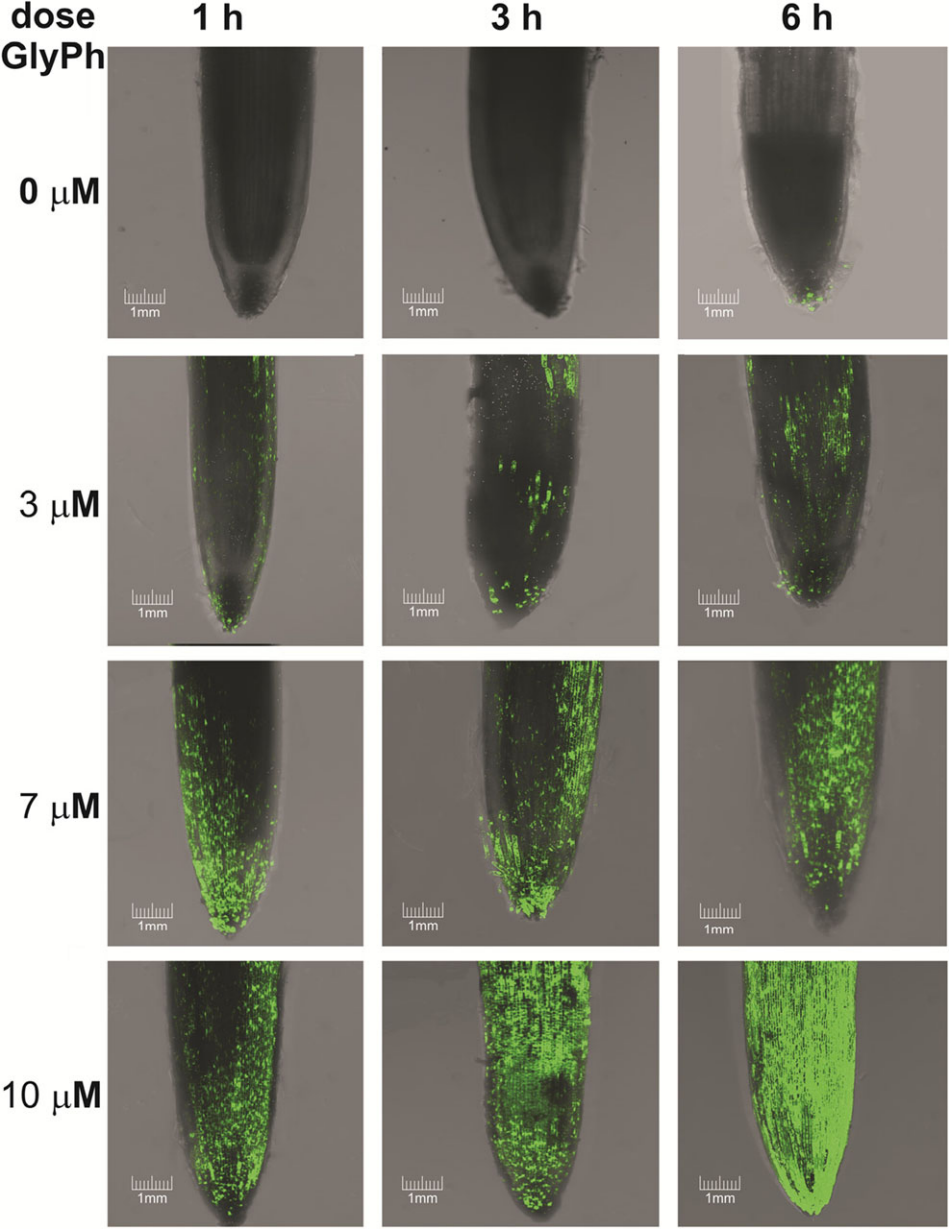
The roots of 9 days old seedlings that were incubated for 1, 3, 6 h in the soil with the addition of 0, 3, 7 and 10 μM concentrations glyphosate (GlyPh). ROS location (green colour) was shown by overlapping confocal microscope images

Reactive oxygen species were detected using non-fluorescent 2′,7′-dichlorodihydrofluorescein diacetate (H2DCFDA), freely penetrating the cells. This compound is deacetylated in the cells and interacts with ROS giving rise to the highly fluorescent 2′,7′-dichlorofluorescein (DCF) which can be detected with a fluorescent microscope. The photographs presented here are complete sets of images from different layers of the specimen, superimposed upon each other (Fig. 6). There were differences in the ROS contents of the apical zones of roots exposed to GlyPh (3, 7 and 10 μM GlyPh for three and 6 h, each), compared with respective controls. GlyPh-induced generation of ROS manifested by DCF fluorescence was observed.

## Discussion

In order to maintain agricultural plant production at a satisfactory level, herbicides are routinely applied. Globally, they account for approx. 40% of all pesticides used for pest control (Grube et al. 2011). In Poland alone, the use of herbicides in 2008 increased three times compared to 2003 (Matyjaszczyk 2011). GlyPh-based wide range herbicide preparations (GBHs - glyphosate-based herbicides) have been used most frequently (Myers et al. 2016). Over the last decade, 6.1 billion kilogrammes of GBHs were applied globally (Grossbard and Atkinson 1985). GBHs are widely used in production of numerous crops, including maize, rape, wheat, barley, beans and soybean (Myers et al. 2016). Grossbard and Atkinson (1985) claimed that GlyPh is practically non-toxic to non-plant life forms such as aquatic and land animals, and humans. Smith and Oehme (1992) also indicated that GlyPh posed no hazard to the environment as, it does not leach into non-target areas and does not accumulate in the food chain. Recently, the opinion on GlyPh has changed due to the research carried out by various research centres (Glyphosate (158) 2005; Bai and Ogbourne 2016). When applied properly, herbicides suppress the development of weeds without significant effect on crop growth. It was found, however, that GlyPh can adversely affect the development of cultivated plants, as reported by Torres et al. (2003), Kohata et al. (2004), Piotrowicz-Cieślak et al. (2010). It poses a particular hazard to young, developing soybean (non-GM) seedlings. The current study demonstrated that GlyPh present in the soil at low concentrations, while not inhibiting soybean seed germination and not restricting the growth of the roots and shoots (Fig. 1), modified the biosynthesis of biogenic amines, particularly LDC and ODC, and thus CAD and PUT contents (Figs 3 and 4).

This study demonstrated that young soybean seedlings take up GlyPh (Fig. 2) from the soil to a very limited degree. The roots, i.e. the organs with a direct exposure to GlyPh, accumulate less herbicide than the shoots. The seedlings only took up 0.52, 0.45, and 0.36% of the dose used, while the remaining part of this dose remained in the soil and would probably decompose with a half-life from 10 to 100 days (47 days on average) (Hornsby et al. 1996). According to Monsanto Glyphosate Half-life in Soil 2014) an average half-life GlyPh is 32 days. The entire plant (the root and shoot together) taken up from the soil (at the highest concentration) 0.60 μg of GlyPh per seedling, while at the lowest concentration, it took up 0.22 μg of GlyPh per seedling.

This proves that the uptake of the active substance increases with an increase in the herbicide content of the soil; however, this increase is not proportional to the active substance content of the soil (Fig. 2). The active substance content of the soil increases by 3.33 times, while in the plant it only increases by 2.72 times. On the other hand, the GlyPh content of the roots increased by 3.3 times, while in the shoots it only increased by 2.5 times. The soybean was cultivated on quartz sand in order to eliminate organic compounds and soil microorganisms that could contribute to the binding and decomposition of GlyPh (Piccolo et al. 1992; Giesy et al. 2000; Zhou et al. 2004). Thanks to the use of quartz sand, we could reasonably assume that all GlyPh present in the substrate is available to soybean roots; however, over 96% of GlyPh remains in the substrate. GlyPh was highly soluble in water (Worthing and Walker 1987; MacBean 2009). The water content calculated based on the difference between fresh mass (Fig. 1b) and dry mass (Fig. 1c) slightly decreased, and the decrease in water content was so insignificant that it did not decrease the plant osmotic potential (Fig. 1d). However, GlyPh was in fact taken up with water. The estimated amount of GlyPh in 1 g of water content in both roots and shoots of the plants treated with the highest dose of herbicide was the same and amounted to 1.2 ng. The same pattern of organ distribution of GlyPh (equal concentrations in roots and shoots) water phase but difference absolute levels was found in plants treated with the other herbicide doses - GlyPh contents were 0.3 and 1 ng/g plant water for soil GlyPh levels 3 and 7 μM, respectively.

Although only very low quantities of GlyPh were detected in soybean seedlings, the herbicide might have acted toxically not only as inhibitor of 5-enolpyruvylshikimate-3-phosphate synthase (its normal target enzyme) but also by disrupting the metabolism BAs. Plants, in order to counteract environmental stresses, accumulate protective compounds, including BAs (Yin et al. 2014). In response to GlyPh, 9-day-old seedlings accumulated CAD and PUT and the shoots accumulated SPD too (Figs. 3 and 4). SPD is found in dry soybean seeds (Ohe et al. 2005), while PUT is dominant in germinating seeds (2-day-old roots) (Glória et al. 2005). In the current study, the roots also contained more PUT and CAD compared to the shoots (Figs. 3 and 4). Generally, the most frequently found amines in plant tissues are PUT, SPD and SPM (Baciak et al. 2017). The BA content is determined by the activity of enzymes involved in the synthesis of these amines. Generally, the activity of studied decarboxylases correlated with BA contents, in particular, a strong relation between the ODC and PUT content was observed (Figs. 3 and 4).

BAs are involved in the control of plant growth (Kusano et al. 2007). Therefore, we carried out the tests on nine-day-old seedlings, that is plants at the phase of intensive growth. Moreover, BAs are involved in the response to both biotic and abiotic stresses (Kaur-Sawhney et al. 2003). Since CAD and PUT contents in the plants increased under the influence of GlyPh in the soil (Figs. 3 and 4), we may assume that they acted as components of plant’s response to stress. The enzymes controlling these BAs are considered the first line of defense against phytotoxic agents of the environment (Van Breusegem et al. 2001; Apel and Hirt 2004). It may therefore, be suspected that the free radicals generated in response to the herbicide are removed by POD and CAT. The function of both enzymes is to participate in the alleviation of oxidative stress (Michiels et al. 1994; Gravato et al. 2005). In this study, CAT activity in the roots and shoots decreased slightly (Fig. 5). Therefore, either relatively small amounts of hydrogen peroxide were generated or the enzyme was deactivated under the influence of GlyPh. It should be noted that this enzyme catalyses the decomposition of toxic hydrogen peroxide, into water and molecular oxygen without generating free radicals (Cerutti et al. 1994; Otto and Moon 1996).

Like CAT, POD is present in all land plants (Passardi et al. 2004; Cesarino et al. 2013) and is involved in numerous physiological and developmental processes in plants, including growth and stress responses (Passardi et al. 2004). It should be emphasized that in soybean roots the activity of POD was six times higher than that of CAT (Fig. 5). This was perhaps so, because POD is an important player in the process of cell elongation, which was very active in the young seedlings under study. In the shoots, POD activity was many times lower, compared to roots.

By using fluorescent microscopy, it was possible to conclusively confirm the increase in root ROS content under the influence of GlyPh (Fig. 6). H_2_DCF-DA is a lipophilic and non-fluorescent compound which freely infiltrates cell interiors (Huang et al. 2016). It could be also be demonstrated that free radicals were generated as early as the first hour after the application of GlyPh and their content increased with the time of plant exposure to the herbicide.

## Conclusion

The obtained results indicate that further research into the effects of glyphosate on young cultivated plants is required. It was demonstrated, the active substance of the herbicide Roundup Ultra 360 SL is taken up by plants in very low quantities. Nevertheless, the effects of this compound on plant metabolism were noted. On the other hand, 96% of the herbicide remained in soil and could potentially have adverse affects on the environment or neighbouring and succeeding plants.
